# Electrochemical lithium extraction from hectorite ore

**DOI:** 10.1038/s42004-024-01378-x

**Published:** 2024-12-03

**Authors:** Andrew Z. Haddad, Hyungyeon Cha, Liam McDonough, Chaochao Dun, Garrett Pohlman, Jeffrey J. Urban, Robert Kostecki

**Affiliations:** 1 Energy Storage and Distributed Resources Division, Lawrence Berkeley National Laboratory, Berkeley, CA USA; 2 The Molecular Foundry, Lawrence Berkeley National Laboratory, Berkeley, CA USA

**Keywords:** Batteries, Batteries

## Abstract

Electrochemical technologies add a unique dimension for ore refinement, representing tunable methods that can integrate with renewable energy sources and existing downstream process flows. However, the development of electrochemical extraction technologies has been impeded by the technological maturity of hydro- and pyro-metallurgy, as well as the electrical insulating properties of many metal oxide ores. The fabrication and use of carbon/insulating material composite electrodes has been a longstanding method to enable electrochemical activation. Here, using real hectorite ore, we employ this technical approach to fabricate hectorite-carbon black composite electrodes (HCCEs) and achieve electrochemical activation of hectorite. Anodic polarization results in lithium-ion release through a multi-step chemical and electrochemical mechanism that results in 50.7 ± 4.4% removal of lithium from HCCE, alongside other alkaline ions. This technical proof-of-concept study underscores that electrochemical activation of ores can facilitate lattice deterioration and ion removal from ores.

## Abstract (web summary)

Electrochemical technologies for ore refinement provide a unique opportunity to integrate with renewable energy sources but are impeded by the insulating properties of many ores. Here, the authors take inspiration from the lithium-ion battery field and fabricate hectorite–carbon black composite electrodes to enhance electron percolation into hectorite enabling lithium-ion release through a multi-step (electro)chemical mechanism.

## Introduction

Historically, ore refinement has been dominated by thermally coupled hydrometallurgical processes or pyro-metallurgy. For example, the Bayer process is used to extract alumina from bauxite, the sulfuric acid roast process^1^ and the limestone gypsum roast (LGR) process^2,3^ are used to extract lithium from spodumene or phyllosilicate ores, respectively; the high-pressure acid leach process (HPAL) is used to extract nickel from laterite nickel oxide ores, and pyrometallurgical processing of both copper and nickel sulfide ores^4^ is used to recover nickel and copper. These types of thermochemical processes have dominated critical material and base metal extraction technologies for over a century, the totality responsible for supplying raw materials that are integral to many technologies used in energy storage applications, aviation, building materials, and consumer products.

As global demand for stationary electrical energy storage and electric transport continues to increase, projected to reach 4600 GWh by 2040^5–7^, so too will the demand for the critical elements needed to power this shift. For example, estimates suggest a need for more than 100,000 metric tons (t) of lithium (elemental) per year by 2025, a 300% increase from 2018 levels^8^, and by 2100 a staggering 413,000–704,000 t of Li per year^9,10^. The trend is similar for copper, nickel, cobalt, phosphate, vanadium, and rare earth elements (REEs)^4^. This dramatic surge in mineral demand complicates efforts to reach net zero targets by the second half of the century further exacerbating the materials production carbon footprint problem^11^, and may lead to profound materials supply implications. Despite the ubiquity of ore processing and its increasing importance in sustaining a clean energy future, they have had little change since their inception.

Recently, lithium reserves were identified near the McDermitt caldera (USA) at Thacker Pass with estimates suggesting ~20–40 Mt (million metric tonnes) of lithium contained within the whole caldera were identified^12^. These reserves are recognized as both Illite-bearing Miocene lacustrine sediments that can have grades up to 1 weight % lithium, and smectite-rich claystones, such as hectorite, and other lithium-bearing claystones that can have up to 0.4 weight % of lithium. These sedimentary Li resources are also found in other areas such as the Mojave Desert, southern Nevada, Mexico, and Serbia^7,13–15^ and are attracting numerous large-scale commercial mining and extraction ventures employing traditional thermal-based LGR processes or concentrated acid leaching^16,17^.

Electrochemical leaching is an emerging field that utilizes electrochemical reactions, electrical fields, or a combination of chemical and electrochemical reactions to facilitate metal ion dissolution from host lattices. Various iterations of electrochemical leaching have been successfully deployed in electronic waste remediation^18–20^, LIB recycling^21,22^, and ore dissolution^23,24^. Compared to chemical and thermochemical approaches, it removes the need for high temperatures or high acid concentrations and can integrate seamlessly with renewable electrons^19^. However, given the heterogeneous nature of the electrochemical reaction and the insulating properties of many oxide-bearing lithium ores, electrochemical leaching strategies are limited by large overpotentials, side reactions, and poor Faradaic efficiency^25^. Various strategies can be deployed to enhance electron transfer kinetics such as the use of soluble chemical promoters that initiate a chemical reaction before electrochemical ion migration^24^, application of acoustic waves^26,27^, or high temperatures to create molten conductive melts.

**Fig. 1:**
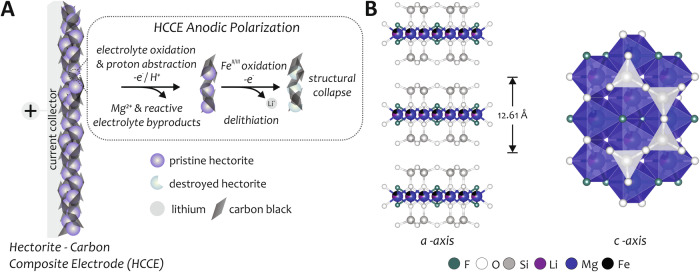
Schematic of electrochemical polarization of a hectorite–carbon black composite electrode, HCCE, and hectorite structure. **A** Polarized aqueous HCCE composites lead to Fe^II/III^ oxidation as well as electrolyte oxidation and generation of reactive byproducts, resulting in ion removal from hectorite and collapse of the hectorite lattice structure. **B** Lattice structure of trioctahedral hectorite consisting of octahedrons, with Li, Mg, and Fe occupying octahedral coordination sites, sandwiched between sheets of SiO_4_ tetrahedrons with an interplanar spacing of 12.61 Å (a-axis and c-axis views shown). These layers repeat with sodium, potassium, and calcium occupying the space between. Interplanar ions are not shown; crystallographic information is available in Table S1.

To this end and in pursuit of developing additional mineral processing technologies, we illustrate a proof-of-concept for electrochemical activation-enabled ion release by utilizing the strategy of mineral ore-carbon composites. Li^+^ (and other alkaline ions) can be electrochemically deintercalated from a variety of inorganic host 2D and 3D mineral materials. In fact, the basic mechanism of the Li-ion rocking-chair battery is grounded in this concept. The fabrication and use of composite electrodes have also been a longstanding method to enable electrochemical activation of insulating materials, most notably demonstrated with olivine and transition metal oxide electrode active materials in lithium-ion batteries^28–30^. We capitalize these two venerable methods and show that anodic polarization hectorite–carbon black composite electrodes (HCCE) achieve ion removal from real hectorite clay via a multi-step mechanism implicating both electrochemical (E) and chemical (C) events, Fig. 1A. An initial oxidation of electrolyte (E) facilitates proton abstraction by surface oxides (C), via proton-coupled electron transfer, resulting in an altered lattice structure. Thereafter, the oxidation of iron (E) results in an oxidative deintercalation, which further alters and weakens the lattice. The weakened lattice structure is then attacked by reactive byproducts generated from electrolyte oxidation (C) leading to structural collapse of the original hectorite lattice, and substantial alkaline ion release into the electrolyte. Our results provide a foundation for the development of effective electrochemical ore extraction strategies.

## Results and discussion

Hectorite is classified as a smectite^31–37^, composed of a sheet of octahedrally coordinated Mg^2+^, Fe^2+^, or Li^+^ ions with oxygen or fluoride as corner-sharing atoms, sandwiched between two identical layers of linked SiO_4_ tetrahedrons^38^. Sheets of this type are superimposed and linked by a plane of cations (Na^+^ K^+^, and Ca^2+^) and water molecules, forming a turbostratic layered material, Fig. 1B^39^. ICP-MS analysis of the hectorite clays used in this study confirms this composition, Table S2. Hectorite, being a phyllosilicate, is a poor conductor in its pristine form making it difficult for electrochemical activation. To alleviate this and improve electron percolation into the material, we prepared a hectorite–carbon black composite electrode (HCCE) to enable electrochemical activation and assess its electrochemical behavior.

**Fig. 2:**
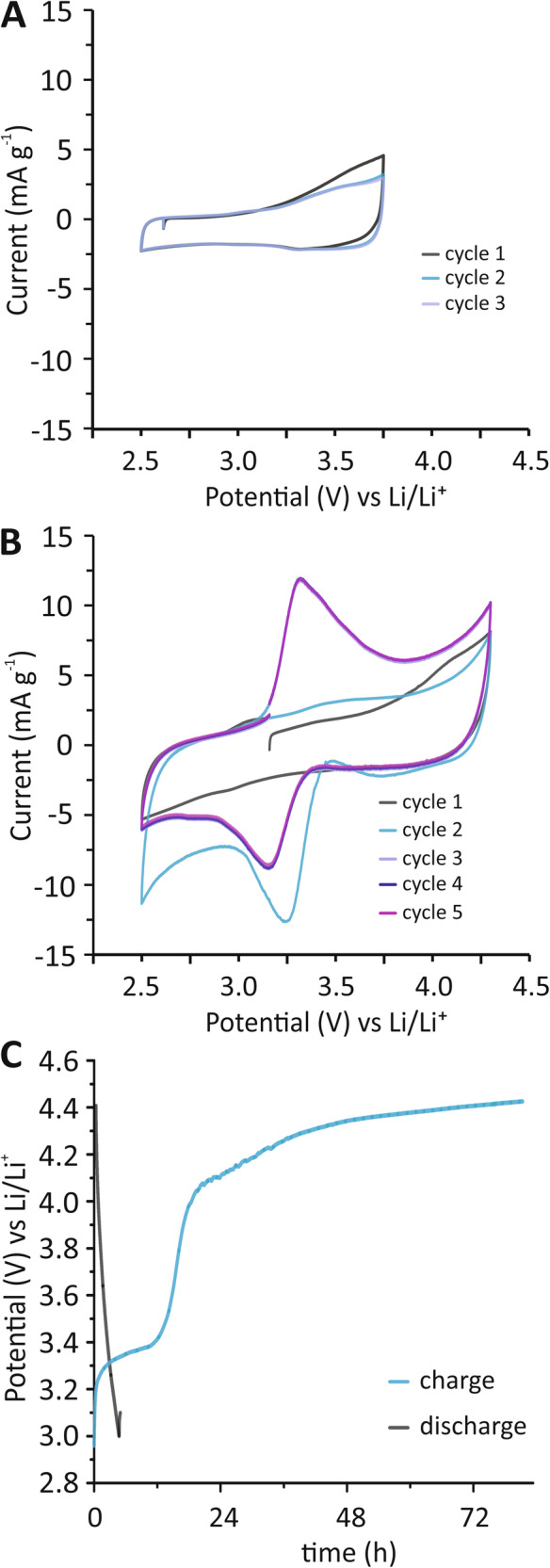
Electrochemical characterization of HCCEs. **A** CVs of HCCE composite electrode cycled three times between 2.5 and 3.75 V showing the first (black), second (blue), and third (purple) cycles. **B** Five CV cycles of HCCE between 2.5 and 4.3 V showing the first (black), second (light blue), third (light purple), fourth (dark blue), and fifth (berry) cycles. **C** Galvanostatic charge (blue) and discharge (black) curves of HCCE at a current density of 0.72 mA g^−^^1^ between 2.9 and 4.5 V. WE: HCCE; CE: Li; RE: Li; Electrolyte: LiPaste generation-2 LIB electrolyte (Tomiyama), see Supplementary Information for full details.

Figure 2A shows cyclic voltammetry (CV) scans of the composite HCCE cycled three times between 2.5 and 3.75 V (vs Li/Li^+^) at a scan rate of 0.5 mV s^−^^1^. Cycle one shows negligible anodic and cathodic current, with only a slight increase in anodic current between 3.5 and 3.75 V. Voltammograms from cycles two and three are similar but show a reduction in the anodic current intensity at 3.75 V. Extending the anodic sweep limit to 4.3 V yields a different set of voltammograms, Fig. 2B. Cyle one shows an irreversible anodic event with a peak potential at 4.3 V, due to electrolyte oxidation, and no cathodic events. Cycle two results in a large irreversible cathodic event with a peak potential of 3.25 V. This species is then observed as a reversible event in cycle three with an anodic peak potential at 3.4 V and cathodic peak potential at 3.2 V. The lack of a cathodic event at 3.25 V in cycle one suggests that both electrolyte oxidation at 4.3 V and subsequent reduction of those reaction products at cathodic potentials below 3 V in cycle one is necessary to condition the structure and enable the electrochemical activity observed in the following cycles. The redox couple seen in cycle three corresponds to the deintercalation of lithium in hectorite and is consistent with the oxidation potential range observed in lithium iron phosphate (LFP) cathodes^40^, suggesting that Fe^2+^ oxidation is implicated in the deintercalation event. We note that there is a possibility of magnesium deintercalation. However, prior work suggests that the insertion/extraction potentials of magnesium from magnesium iron oxide cathode materials are significantly lower than those observed in this work^41^. CVs were performed using a 100% carbon-based electrode and showed a voltammogram typical of a capacitive system, Fig. S1, confirming that the reversible redox character is attributed to hectorite. Cycles four and five are nearly identical to cycle three, suggesting that the conditioning of the electrode is complete after three cycles. The observed order of electrochemical events would suggest that the oxidation state of iron in hectorite is Fe^3+^. However, the anodic charge consumed in cycle three, 36.93 mC, is higher than in the preceding cathodic process in cycle two, −26.2 mC, Fig. S2. This suggests that there is a mixed valence state of iron in hectorite, with an apparent ratio of Fe^2+^ to Fe^3+^ of 0.41–1. In fact, iron valency in smectite clays has been reported to be either solely Fe^3+^ or Fe^2+^, or a mixture of the Fe^2+/3+^^42–44^.

The apparent conditioning of the structure by electrolyte oxidation and subsequent reduction of these reaction products at cathodic potentials below 3 V may be related to two possible mechanisms: (i) proton generation from residual water in the electrolyte (<10 ppm) and surface etching of MgO in hectorite, or (ii) the generation of protons and other reactive moieties from organic carbonate solvents oxidation. Organic carbonate-based electrolyte degradation mechanisms have been extensively evaluated previously and suggest a two-step reaction mechanism, proton abstraction from ethylene carbonate (EC) and transfer to the surface oxygen sites, followed by a ring opening of the proton abstracted EC to generate reactive oxalates and or other chelating moieties^45–48^. It appears that the electrolyte oxidation at 4.3 V is essential for the electrochemical activation of hectorite and the emergence of the reversible Fe^2+/3+^ redox couple. Protons generated by electrolyte oxidation can react with edge MgO surface sites in hectorite, a process that is known to be highly exergonic^3,49^, resulting alteration of Mg^2+^ ion in the octahedral sites adjacent to the surface. Such processes would alter the lattice at the surface to open the appropriate channels for subsequent lithium-ion deintercalation.

After five CV cycles of the electrode, Galvanostatic cycling of HCCE was performed between the open circuit potential (2.9 V) and 4.5 V at *i* = 0.72 mA g^−^^1^. The anodic polarization curve yields a potential profile that shows a distinct plateau at 3.4 V, Fig. 2C, consistent with the anodic peak observed in the CV of the HCCE. A second plateau is observed at 4.3 V corresponding to the electrolyte oxidation event observed at 4.3 V in the CVs of HCCE. We note the anodic polarization curve resembles those observed for LiFePO_4_ lithium-ion positive electrodes^50–52^. In comparison to the HCCE polarization curve, a pure carbon electrode control, Fig. S3, shows capacitive behavior with no plateaus, confirming iron oxidation and ion deintercalation as the primary anodic reaction in the HCCE. The following cathodic discharge polarization profile of HCCE, Fig. 2C, shows markedly different features in both shape and duration. There is a rapid voltage decrease that resembles a pseudocapacitive or capacitive discharge behavior^53–61^. This illustrates that the anodic charging process irreversibly alters hectorites structural ordering, preventing the possibility of ion re-intercalation. Reversible Li intercalation and deintercalation into analogous layered iron phyllosilicates have been previously reported^62^. This reversibility is attributed to the charge-neutral sheets in the layered iron phyllosilicate. The irreversible behavior observed here is consistent with this as hectorite does possess interplanar cations. We also note that irreversible chemical redox behavior and structural degradation of similar smectite clays have been previously observed^63^.

To interpret the Faradaic efficiency (FE) of the process we performed chronoamperometry. FE is a factor to evaluate the effectiveness of an electrochemical reaction and evaluates the usage of electrons passed through a cell. It is defined as the amount of electrons used for the intended product formation relative to the theoretical amount of product that can be produced from the total charge passed^64^. To deconvolute the charge obtained from the oxidation of the active material (HCCE) from the charge obtained from the oxidation of the electrolyte, we applied a potential of 4.5 V vs Li^+^/Li with HCCE as the positive electrode (after CV cycling five times) and then again at the same potential using a pure carbon black electrode. Chronoamperometry experiments were left to run until the current reached zero. An overview of the calculation methodology and plots of charge are shown in Fig. S4. The results show that the FE of the process is 54.8%, suggesting that electrolyte oxidation is an active participant in the reaction.

During the anodic polarization, there is a phase change event and irreversible alteration to the structural order of the hectorite lattice. As mentioned previously, we hypothesize that this may be due to the consumption of protons from EC oxidation and alteration of magnesium surface sites in hectorite, followed by electrochemically coupled iron oxidation and lithium-ion deintercalation. Additionally, given that there appears to be some meaningful level of electrolyte oxidation at 4.3 V, oxidized electrolyte byproducts may also be reacting chemically with hectorite, further exasperating structural enervation. Such induced structural defects and lattice perturbations could promote fast ion diffusion as well as trigger an irreversible structural transformation of the clay lattice structure. Indeed, the capacitive and rapid cathodic discharge behavior of HCCE, Fig. 2C, supports this hypothesis.

**Fig. 3:**
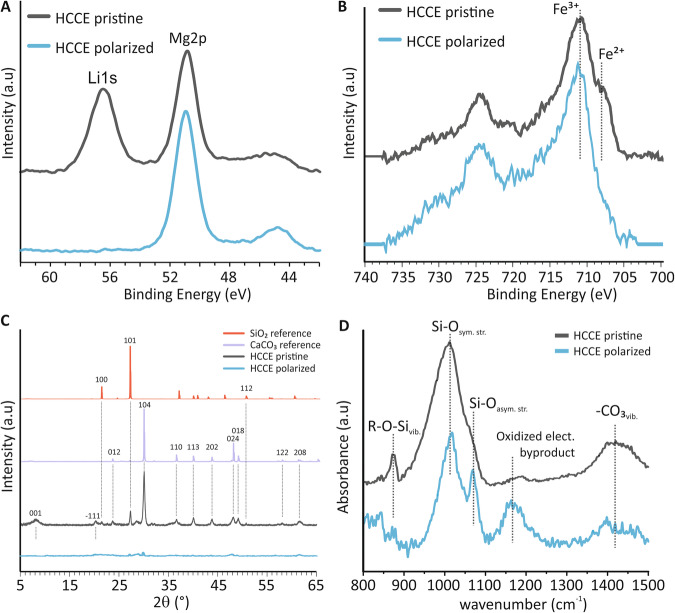
Structural and morphological analysis of pristine and polarized HCCEs. **A** Li1s high-resolution XPS spectra pristine (black) and polarized (blue) HCCE’s. **B** Fe2p high-resolution XPS spectra pristine (black) and polarized (blue) HCCE’s. **C** Diffraction patterns of pristine HCCE (black), and HCCE after Galvanostatic polarization (blue); SiO_2_ (red) and CaCO_3_ (purple) references. **D** ATR-FTIR spectra pristine HCCE (black), and polarized HCCE (blue); R = Li^+^, Mg^2+^, or Fe^2+^.

High-resolution XPS reveals a change in the lithium and iron environments of hectorite after polarization. The polarized HCCEs show no Li1s peak at 56.6 eV in comparison of pristine HCCE, Fig. 3A, which does exhibit the Li1s core level peak. This is consistent with lithium-ion presence as one of the predominant octahedral cations in trioctahedral type smectites^37^. Depth-penetrating Li1S XPS provided additional information about the bulk lithium environment. As seen in Fig. S5, probing below the surface of the electrode reveals a gradual increase in the Li1s core level at 56.6 eV, suggesting that there is still considerable lithium remaining in the HCCE after polarization. Other alkaline ion spectra were examined but revealed little change when comparing pristine to polarized samples, Fig. S6. XPS analysis of the transition metals present in hectorite was also performed to learn more about the nature of electron transfer during polarization. There are salient differences between Fe2p high-resolution XPS spectra of pristine and polarized HCCEs, Fig. 3B. The pristine spectrum shows two distinct peaks with binding energies of ~711 and 708 eV corresponding to oxidation states of Fe^3+^ and Fe^2+^. After polarization of HCCE, only the peak at a binding energy of ~711 eV is present, suggesting that all Fe^2+^ has been oxidized to Fe^3+^^65,66^. Depth-penetrating XPS, Fig. S7A, and electron energy-loss spectroscopy (EELS), Fig. S7B, further confirmed that the oxidation state changes are a bulk phenomenon rather than a surface artifact. EELS Fe L edge spectra, Fig. S6B, of the pristine material show energy losses in the Fe L-3 regions with two peaks related to Fe^2+^ and Fe^3+^ while the polarized samples exhibit an L edge region with only one peak, consistent with Fe^3+^. The EELS and XPS results corroborate our earlier hypothesis of a mixed iron valency in pristine hectorite and confirm that iron oxidation is implicated in the release of ions after polarization. Other notable changes to the HCCE are seen in high-resolution C1s spectra, Fig. S8A, B, showing a high degree of carbon oxidation in the composite material after anodic polarization. Collection of additional high-resolution Mn2p, and Ti2p, minority components of hectorite observed in our ICP-MS measurements, scans show no signal changes upon electrochemical polarization, suggesting that iron is the only transition metal implicated in the redox activity of the HCCEs.

XRD, FTIR, and FIB-SEM provide additional bulk characterization information that confirms the structural deterioration of hectorite during anodic polarization. X-ray diffraction patterns of pristine HCCE show 001 reflection at 7° 2θ, Fig. 3C, arising from the interplanar spacing of 12.61 Å between hectorite’s silicon layers^14,67^, in addition to a −111 reflection at 20° 2θ. In large part, however, the XRD pattern is dominated by CaCO_3_ (calcite) and SiO_2_ (quartz), both of which commonly co-occur as admixtures amongst hectorite. Diffraction patterns collected after galvanostatic polarization of the hectorite HCCEs show noticeable divergence as these are amorphous, confirming the deterioration and structural collapse of the clay lattice as well as the other mineral phases. High-resolution transmission electron microscopy (HR-TEM) was also performed to confirm the amorphous nature of the leached electrode, Fig. S9. This is consistent with electrochemical data which implicates all three octahedral metal ions in the mechanism. These lattice perturbations ostensibly serve as the impetus for structural reformation.

Attenuated total reflectance Fourier transform infrared spectroscopy (ATR-FTIR) of the HCCE before and after galvanostatic polarization further supports the observed structural collapse and provides additional insight into the chemical deterioration of the clays resulting from reactions with reactive oxidized electrolyte intermediates, Fig. 3D. Pristine HCCE features three prominent bands. All of the bands, 870, 1010, and 1400 cm^−^^1^, have been previously assigned as the vibration of metal-O octahedral^68^, Si-O symmetric stretching^69^, and CO_3_^2^^−^ vibration of calcium carbonate^70^, respectively. After anodic polarization, there are stark changes in the IR spectrum. The metal-O octahedral stretching band at 870 cm^−^^1^ is removed, implying that the octahedrally coordinated ions are considerably altered. Interestingly, there is the emergence of two new vibrational modes at 1090 and 1160 cm^−^^1^. The band at 1090 has been observed in the literature previously, assigned to the asymmetric Si-O stretches that result from structural lattice alteration after treatment of hectorite with 1.5 M acids^68,69^. This assignment suggests that similar chemical and structural degradation occurs when subjecting hectorite to anodic polarization. SEM images of polarized HCCE further support this. After anodic polarization, there is substantial morphological alteration and an overall reduction in the size of hectorite particles, transforming into smaller, porous particles, Fig. S10A, B. The band at 1160 cm^−^^1^ can be attributed to possible C–O stretching associated with organic electrolyte decomposition products on the surface of hectorite.

The EC-rich solvent used herein (see “Methods” section) suggests that the oxidized electrolyte byproduct identities could include oxalates, di-carbonyls, and carbonates^45,46^. First-principles calculations have indicated that carbonate ester solvents are oxidized at high potentials via direct electron transfer to produce protons, independent of the electrode surface^45–48^. Moreover, protons and these oxidized electrolyte byproducts have a high propensity to chemically react with metal oxide in the electrode to induce further structural deterioration and promote additional ion removal beyond transition metal coupled oxidation and deintercalation^46,48^. To confirm this, we probed the MgO and Fe_2_O_3_ environments in both pristine and polarized HCCE using FTIR between the range of 460 to 640 cm^−^^1^, Fig. S11. There are changes in the vibrational modes of the polarized spectrum between 460 and 520 cm^−^^1^, consistent with the removal of MgO. Pristine samples show characteristic stretching vibrations of Fe_2_O_3_ between 580 and 620 cm^−^^1^. After polarization, the bands are slightly shifted in addition to the emergence of a new band at 590 cm^−^^1^. Collectively, the results suggest that chemical reactions between protons or oxidized electrolyte byproducts and both magnesium and iron oxides are implicated in the leaching mechanism.

**Fig. 4:**
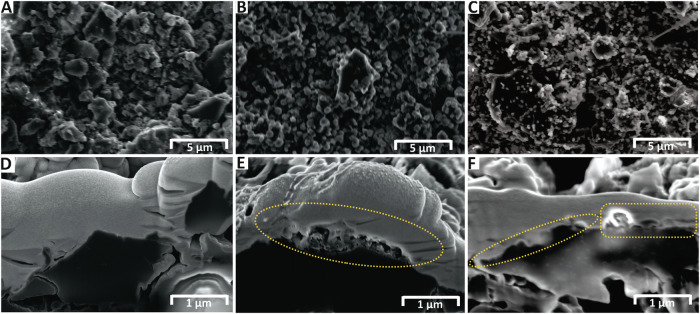
FIB-SEM analysis of HCCEs before and after galvanostatic anodic polarization. **A** SEM image of pristine HCCE. **B** SEM image of polarized HCCE held at 4.5 V for 1 h. **C** SEM image of polarized HCCE held at 4.5 V for 6 days showing enhanced surface film formation. **D** Cross-sectional FIB analysis of pristine HCCE (50,000× magnification). **E** Cross-sectional FIB analysis HCCE polarized at 4.5 V for 24 h showing surface film layer formation on the surface of the electrode (50,000× magnification). **F** Cross-sectional FIB analysis of polarized HCCE at 4.5 V for 6 days showing large surface film (50,000× magnification).

FIB-SEM analysis shows the formation of a surface film on HCCE after anodic polarization, supporting the hypothesis of oxidized electrolyte byproduct film formation. SEM images show hectorite in pristine HCCEs to be 1–2 µm in diameter with smooth surfaces, Fig. 4A and Fig. S12A. This contrasts with HCCEs held at 4.5 V for 24 h, Fig. 4B, and 144 h, Fig. 4C, that both show particle surfaces covered with spherical nodules <1 µm in size and non-uniformly distributed across the surface, yet becoming noticeably more heterogeneous after longer polarization time of 144 h. FIB analysis shows the formation of surface films on the polarized electrodes, as demonstrated by the deposits and gaps (yellow dotted circles) under the sputtered coating. Conversely, the pristine HCCE samples show a uniform coating with no apparent inconsistencies, Fig. 4D–F. Nanopore formation is also confirmed by FIB analysis showing clear nanopore morphology in the polarized samples, contrasting with images of the pristine sample, Fig. S12B.

To probe the ion release from HCCE and to quantify the effectiveness of ion removal, ICP-MS analysis of the pristine and polarized HCCEs was performed. Table S3 provides an overview of the changes and conclusively shows that calcium, magnesium, lithium, sodium, and iron are all released from the HCCEs after polarization. This confirms that the anodic polarization of HCCE results in a complete dissolution of the hectorite lattice. Along with the removal of lithium, 50.7 ± 4.4%, substantial levels of calcium, sodium, and magnesium are released from the electrodes, 79.6 ± 4.5%, 73.6 ± 2.6%, and 70.9 ± 2.5%, respectively. The high level of release of these ions is not surprising given that they constitute the main intraplanar ions in hectorite. These results support the stark structural changes observed in XRD, ATR-FTIR, EELS, and XPS data and suggest that anodic charging is an effective method for the mineral dissolution of ores.

The prospect of eliminating the use of a kiln during lithium extraction from sedimentary clays, which necessitates the use of vast amounts of chemical reagents and temperatures of 1000 °C^2^, is intriguing. To provide context for how such electrochemical dissolution technologies could be competitive with incumbent industrial methodologies we have performed a CO_2_ and energy intensity estimate of the LGR process to ascertain energy and CO_2_ intensity baselines. Mining operations employing the LGR process for lithium removal from clays report that the extraction facility, which encompasses the kiln and other reagents required for leaching, represents 72% of process plant Capital costs. Additionally, the extraction facility accounts for 72% of the total energy consumed, or 12.1 MWh per t of lithium carbonate equivalent (LCE)^71^. From a carbon intensity perspective, the extraction facility accounts for between 4.4 and 14.1 t of CO_2_ per t of LCE, dependent on the electricity source. Considering the use of natural gas, it is 6.83 t of CO_2_ per t LCE, the majority of this, 5.47 t of CO_2_, is attributed to power consumption while the remaining 1.36 t of CO_2_ per t of LCE is attributed to reagent use (see Table S4 for full details). We note that these numbers consider only the extraction step and not the additional minority energy-consuming processing steps that afford battery-grade Li_2_CO_3_ or LiOH-H_2_O. These estimates provide a suitable context for what would be competitive with incumbent technologies in terms of both energy and CO_2_ intensity.

## Conclusion

We have described an electrochemical approach for the liberation of lithium and other alkaline ions from hectorite clay using anodic polarization. Mechanistically, the process follows four steps: two electrochemical, and two chemical. Initially, electrolyte oxidation near 4.3 V facilitates proton abstraction or chemical attack from oxidized electrolyte moieties by surface oxides altering the lattice structure (E, C)^46^. Then, the oxidation of iron at 3.4 V results in an oxidative deintercalation (E), which further enervates the lattice structure. Given the high level of magnesium ion release after polarization, ostensibly, there are additional chemical reactions between the altered lattice structure and oxidized electrolyte (C)^46–48^. The latter represents a chemical mechanism of hectorite decomposition accompanied by lithium and other alkaline ion removal, that supplements the initial ion removal based on transition metal electrochemical oxidation and deintercalation. EC is known to be oxidized at high potentials generating reactive moieties after proton abstraction and ring opening^46–48^. Thermodynamically, these reactive species can react favorably with metal oxides, such as hectorite, liberating additional metal ions and deteriorating the hectorite structure, eventually resulting in a structural collapse of the lattice.

Even in this nascent stage of technological development, there are clear advantages of electrochemical ore processing over incumbent approaches. Foremost is the ability to perform ion extraction at room temperatures, eliminating the need for costly and CO_2_-intensive kiln operation. Second, minimal transition metal content is leached from the ore, which will reduce reagent use associated with downstream leachate purification. Finally, this approach offers a straightforward method to couple ore refinement with renewable electricity sources, a challenge that persists with incumbent technologies. While the technical approach presented here is not suitable for real-world implementation, its translation into more practical aqueous electrolyte systems using slurry composite electrodes may offer a method for ore leaching that could be performed at room temperature. The feasibility and performance of this aqueous electrochemical leaching strategy is the subject of ongoing work. Still, the results presented here are encouraging, opening a new door for the development of electrochemical leaching technologies.

## Methods

### Materials

Polyvinylidene fluoride (PVdF), dimethyl carbonate (DMC), and N-methyl-2-pyrrolidone (NMP) were purchased from Sigma Aldrich. Hectorite was supplied from the Rio Tinto-run US Borax Mine in Boron, CA, and carbon black (Vulcan XC-72R) was purchased from the Fuel Cell Store. T-cells were purchased from Swagelok and insulated with mylar films purchased from Heliumtech. LiPaste generation-2 LIB electrolyte was purchased from Tomiyama.

### Preparation of HCCE electrodes

Hectorite–carbon composites were prepared by ball milling hectorite and carbon black (1:1) for 30 min, and then adding to a 2 weight % polyvinylidene fluoride (PVdF) (Sigma) N-methyl-2-pyrrolidone (NMP) (Sigma) solution to create a viscous slurry. The composite slurry was then doctor-bladed onto an aluminum foil current collector and dried in a vacuum oven at 120 °C for 48 h affording a thin film hectorite–carbon composite electrode (HCCE) with a 10 µm thickness and an active material loading of 7 mg cm^−^^2^. Electrodes were then cut from the thin film sheets using a hole punch and then used as electrodes in T-cells for polarization experiments.

### XRD analysis

Determination of the bulk crystalline mineral composition of hectorite powder, pristine HCCE electrode, and a polarized (after galvanostatic polarization) HCCE was obtained from powder XRD patterns using a Bruker diffractometer with a Cu source. Diffraction patterns were collected from 5 to 70° 2θ with 0.01 frames per 0.5 s at a rotation of 5 rpm. Data was then fitted using diffract EVA software.

### XPS analysis

Pristine and polarized (post-Galvanostatic polarization) HCCE were analyzed by XPS vertical analysis. After electrochemical polarization experiments, the polarized HCCEs were soaked in dimethyl carbonate for 5 min to remove any residual electrolyte and other potential surface layers that may have formed during polarization. Both pristine and polarized HCCEs were then dried in a vacuum oven at 90 °C for 48 h to remove any residual moisture. The samples were then mounted onto a sample holder and 3 points were chosen for analysis. Spectra were collected using a Thermo-Fisher K-Alpha Plus XPS/UPS employing a monochromatic Al X-ray source (1.486 eV). Li1s, Na1s, Mg1s, O1s, C1s, Fe2p, Si2p, Ti2p, Mn2p, C1s, and Ca2p high-resolution spectra were then collected. The spectra were acquired using a spot size of 400 µm and constant pass energy. A combined low-energy electron/ion flood source was used for charge neutralization. A dual monoatomic and gas cluster Argon ion source for depth profiling and sample cleaning. The spectra were referenced to the adventitious carbon reference peak at 284.8 eV and were analyzed using Casa XPS software. Baseline corrections were performed using the Shirley background subtraction. Experiments were performed in triplicate using three different polarized and pristine HCCE samples.

### ATR-FTIR analysis

Hectorite powder and powders of pristine and polarized HCCEs were analyzed using a Thermo Electron Nicolet 5700 FTIR with ATR attachment with diamond crystal. The powder was placed on the crystal and covered with a glass slide and pressed to a constant pressure. Spectra were then collected and processed using Omni software.

### SEM-EDX and SEM-FIB analysis

A FEI Phenom tabletop scanning electron microscope was used to obtain high-resolution images of pristine HCCE and polarized HCCE. Cross-sectioned images were obtained by using the FIB-SEM technique (Helios G4 UX dual beam, FEI). Pt was used to make a protective layer for the samples before milling, and Ga^+^ ions were used for milling.

### ICP-MS analysis

Samples of hectorite, pristine HCCE, and polarized HCCE (after galvanostatic polarization at 3.57 mA g^−^^1^) were digested using an acid microwave digestion and analyzed by ICP-MS, *n* = 3. Polarized HCCE samples were washed with copious amounts of DMC to remove any residual electrolytes and to dissolve any potential surface films that formed during the charging process.

### Electron energy-loss spectroscopy (EELS) analysis

Pristine and polarized samples were analyzed by Electron energy-loss spectroscopy (EELS) based on the 200 kV FEI monochromated F20 UT Tecnai with an energy resolution of 0.5 eV.

### Electrochemical characterizations of pristine and polarized HCCEs

T-cells (Swagelok) insulated with mylar films (Heliumtech) employing an electrode area of 1 cm^2^, were assembled in an argon-filed glovebox with O_2_ and H_2_O content less than 0.1 ppm. Cathodes were the previously prepared HCCE electrodes, and the anode and reference electrodes were lithium metal, and fiberglass paper was used as the separator. The electrolyte was LiPaste generation-2 LIB electrolyte (Tomiyama), a mix of ethylene carbonate and ethyl methyl carbonate (1:1 wt ratio) with 1.2 M LiPF_6_. 50 µL of electrolyte was added for each cell. The electrochemical performance was measured galvanostatically in a voltage window of 2.9–4.5 V using a BioLogic potentiostat at current densities of 0.72 mA g^−^^1^. Five cycles of cyclic voltammograms of pristine HCCE were taken at a scan rate 0.5 mV s^−^^1^ before galvanostatic polarization.

## Supplementary information

Supplemental Information

Description of Additional Supplementary Files

Supplemental Data 1

## Data Availability

The data that support the findings of this study are available within the main text and Supplementary Information.
