# Supplementary material for: Electrochemical lithium extraction from hectorite ore
Source: Commun Chem. 2024 Dec 3;7:285. doi: 10.1038/s42004-024-01378-x (PMC11614861; doi:10.1038/s42004-024-01378-x)
Supplement: Supplementary file 1 — Supplemental Information [file 42004_2024_1378_MOESM1_ESM.pdf]

Supplementary Information for:

Electrochemical Lithium Extraction from Hectorite Ore

Andrew Z. Haddad[1]*, Hyungyeon Cha[1], Liam McDonough[1], Chaochao Dun[2], Garrett Pohlman[1], Jeffrey J. Urban[2], Robert Kostecki[1]*

Correspondence to: azhaddad@lbl.gov and r_kostecki@lbl.gov

This PDF file includes:

Table of Contents

Supplementary Figures

Fig. S1. CVs of 100 % carbon electrode ……………………………………...………**S2**
Fig. S2. Charge integrations of HCCE anodic and cathodic events. …………...……….**S3**
Fig. S3. Galvanostatic polarization curve for carbon electrode…………………............**S4**
Fig. S4. Chronoamperometry of HCCE and a pure carbon black electrode………………**S5**
Fig S5. Depth Li1S XPS of pristine and polarized HCCE……………………………….**S6**
Fig. S6. High resolution XPS spectra of pristine and polarized HCCE ………...............**S7**
Fig. S7. Depth Fe2p XPS and EELs analysis of pristine and polarized HCCE……..........**S8**
Fig. S8. Fitted high resolution C1s XPS spectra………………..……………….……........**S9**
Fig. S9. Pristine and polarized HCCE SEM images……………………………………**S10**
Fig. S10. HR-TEM of pristine and polarized HCCE……………………………………**S11**
Fig. S11. FTIR spectra of polarized and pristine HCCE between 460 and 640 cm^{-1}…….**S12**
Fig. S12. Pristine and polarized HCCE SEM-FIB images……………….…………….…..**S13**
Fig. S13. HCCE GCPL charge discharge curves with 4.0 V charge limit………………**S14**

Supplementary Tables

Table S1. Crystal structure parameters of hectorite……….……………………………**S15**
Table S2. ICP-MS elemental composition of hectorite……………………………........**S15**
Table S3. ICP-MS quantification of pristine and polarized HCCEs……………………**S15**
Table S4. Energy and CO_2 intensity of incumbent LGR process………………………**S16**
References……………………………………………………………………………...**S16**

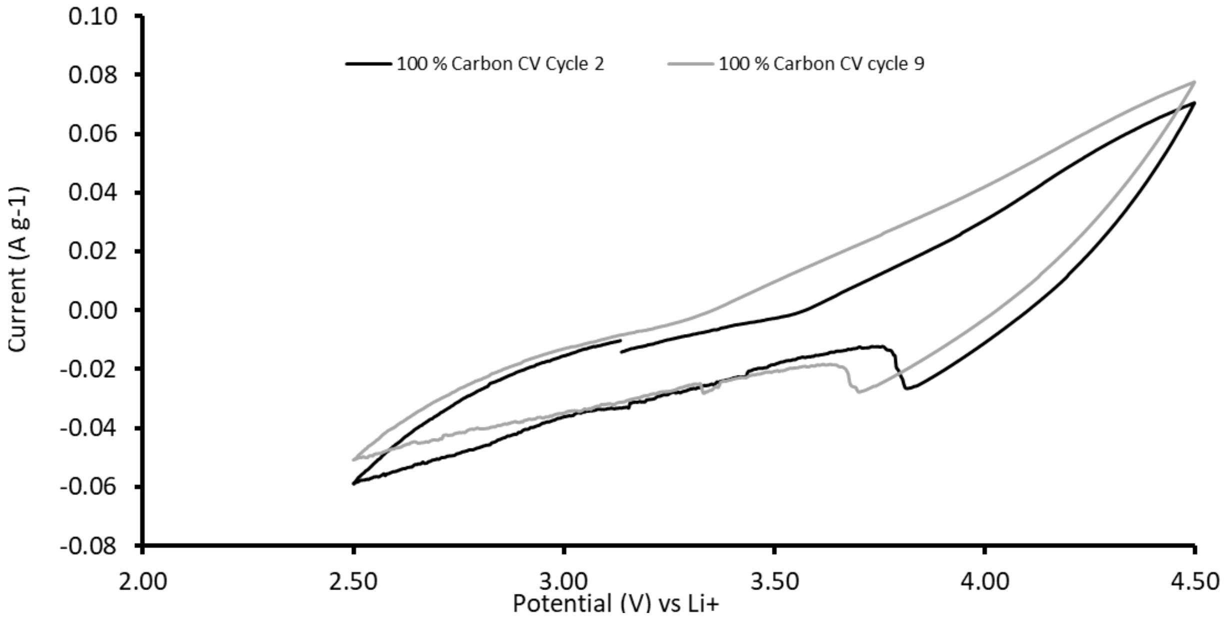

Fig S1. CVs of 100 % carbon electrode. Carbon electrode cycled 9 times at a scan rate of 0.5 mV/s showing cycle 2 (black) and 9 (grey).

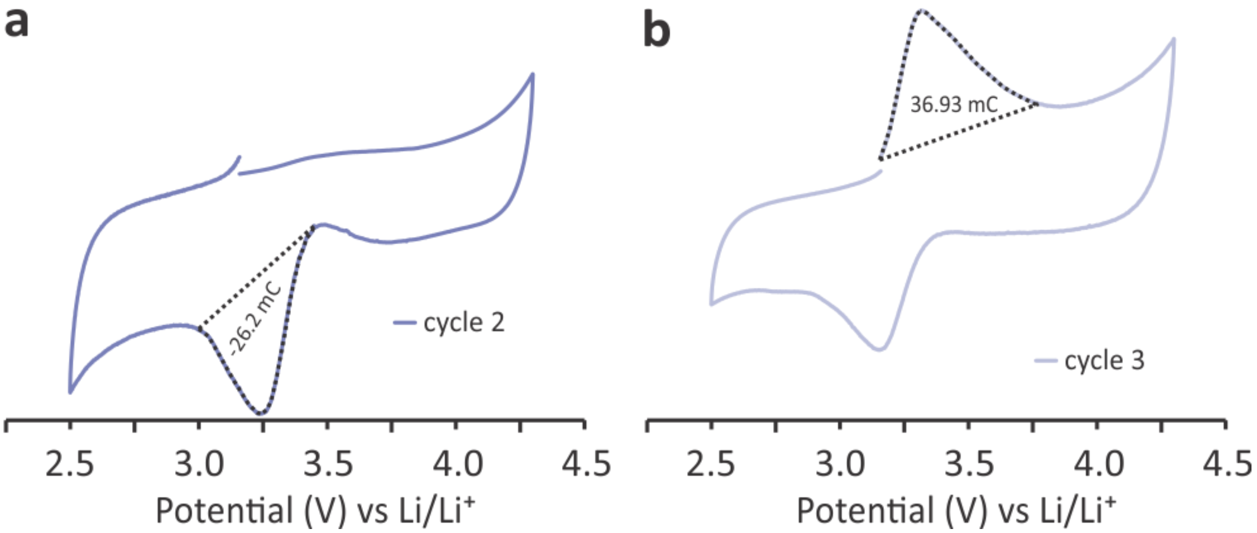

Fig. S2. Charge integrations of HCCE anodic and cathodic events. Integration of charge for cathodic event from cycle 2 (**a**) and anodic event from cycle 3 (**b**).

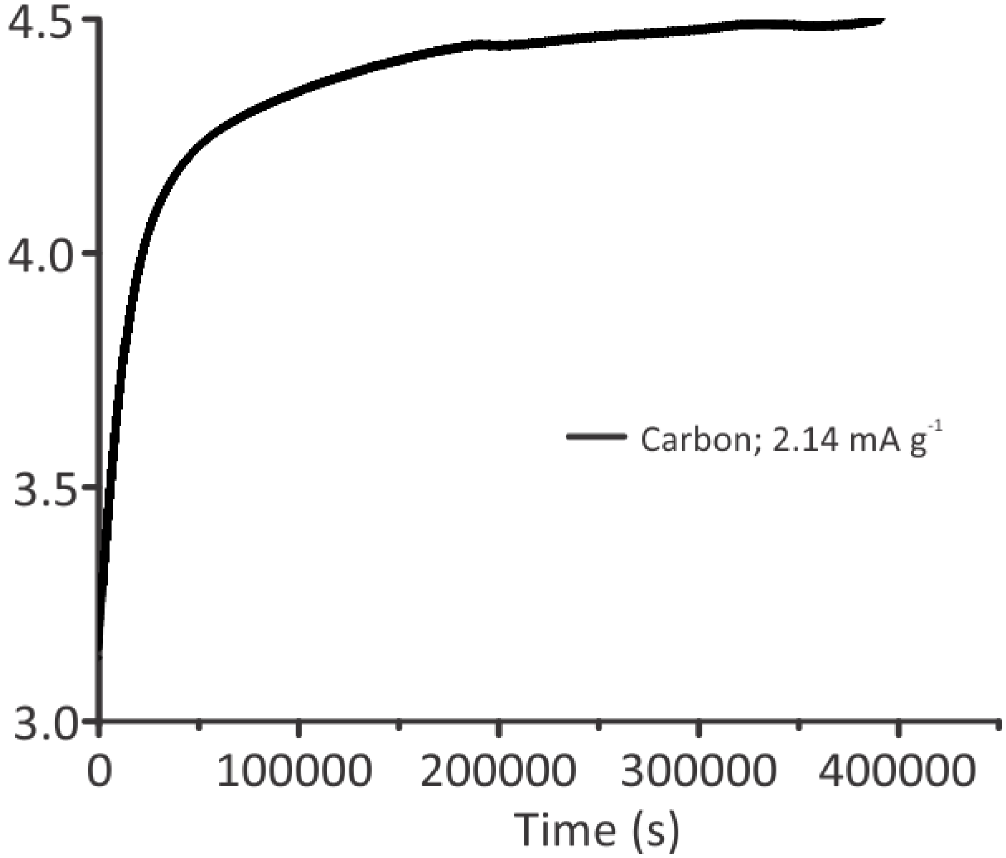

Fig. S3. Galvanostatic polarization curve for carbon electrode. GCPL collected at i = 2.14 mA g^{-1}.

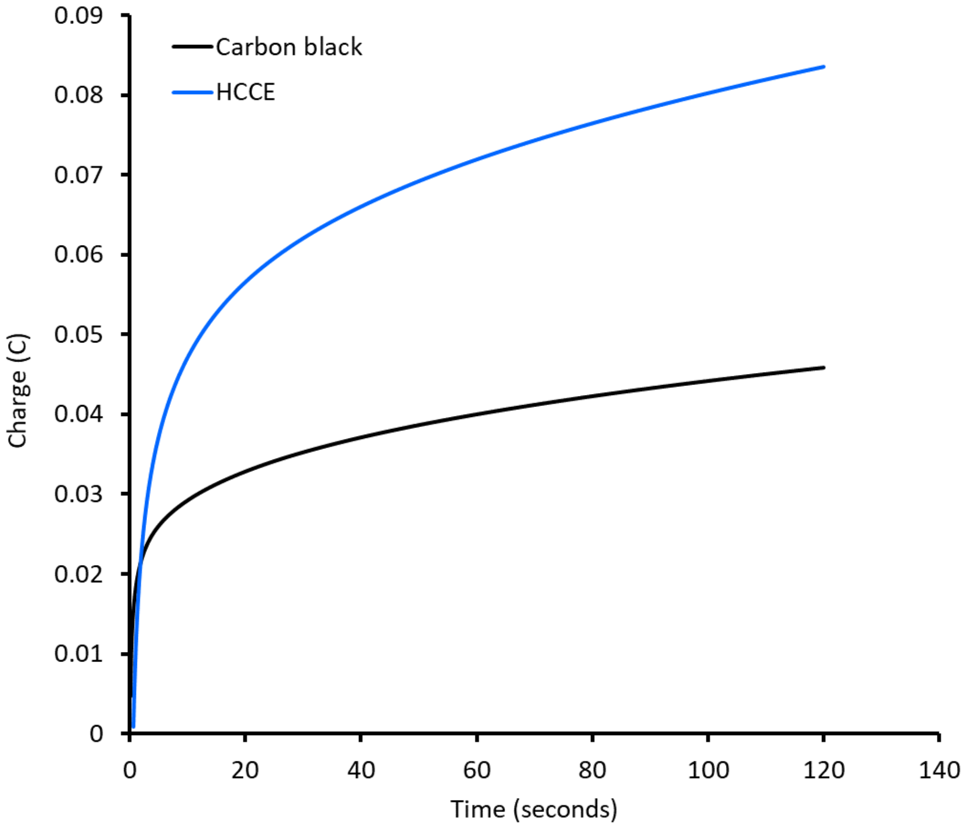

Fig. S4. Charge vs potential for Chronoamperometry of HCCE and a pure carbon black electrode polarized at 4.5 V vs Li^+/Li in 50 µL Gen-2 electrolyte.

To determine the Faradaic efficiency, we used the approach from Marcandalli et al[1,2]. A potential of 4.5 V vs Li^+/Li potential was held until the cell current reached zero for both a pure carbon black electrode (black) and the HCCE composite (blue) in 50 µL gen-2 electrolyte. The total charge from each individual chronoamperometry experiment can be expressed as $Q_{leaching}$ + $Q_{electrolyte\ oxidation}$ (blue) and $Q_{electrolyte\ oxidation}$ (black). The Faradaic efficiency is calculated by equation 1:

$$FE = \frac{Q_{leaching}}{Q_{leaching} + Q_{electrolyte\ oxidation}} \quad (1)$$

$$FE = 0.083582 / 0.129421$$

$$FE = 0.6458 \text{ or } 64.58\%$$

There is 1.01 % Fe(II) in the sample or 18.1 mmoles. We observe 51% lithium extraction or 15.4 mmoles, which would account for 85% of the charge attributed to $Fe^{2+/3+}$ oxidation and consequential lithium deintercalation. Thus, 0.85 x 0.6458 (from Figure S4) = **54.8 true Faradaic efficiency for lithium extraction.**

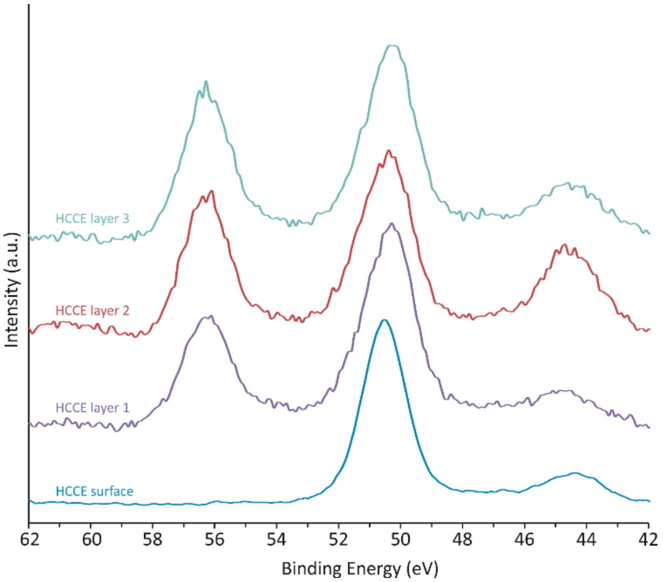

Fig S5. Depth Li1S XPS of pristine and polarized HCCE. (a) High resolution Li1s XPS depth profile spectra of HCCE.

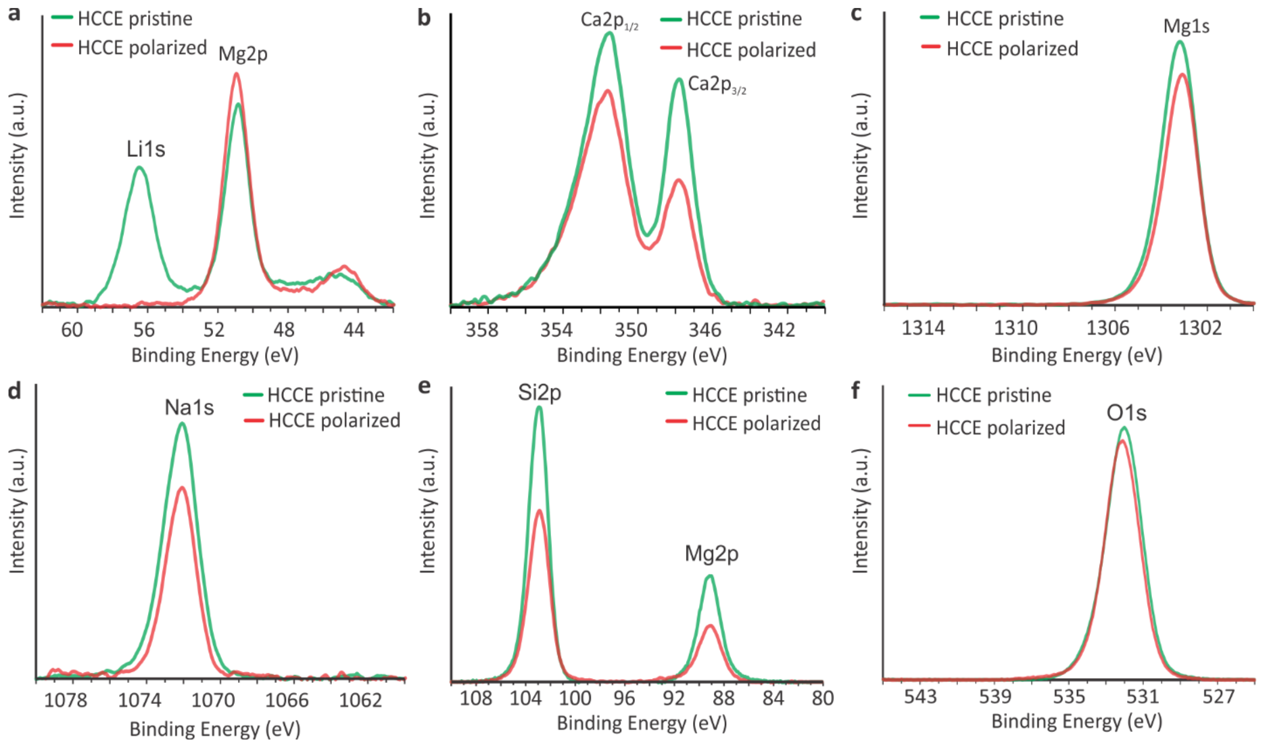

Figure S6. High resolution XPS spectra of pristine and polarized HCCE. (**a**) Li1s. (**b**) Ca2p. (**c**) Mg 1s. (**d**) Na1s. (**e**) Si2p. (**f**) O1s

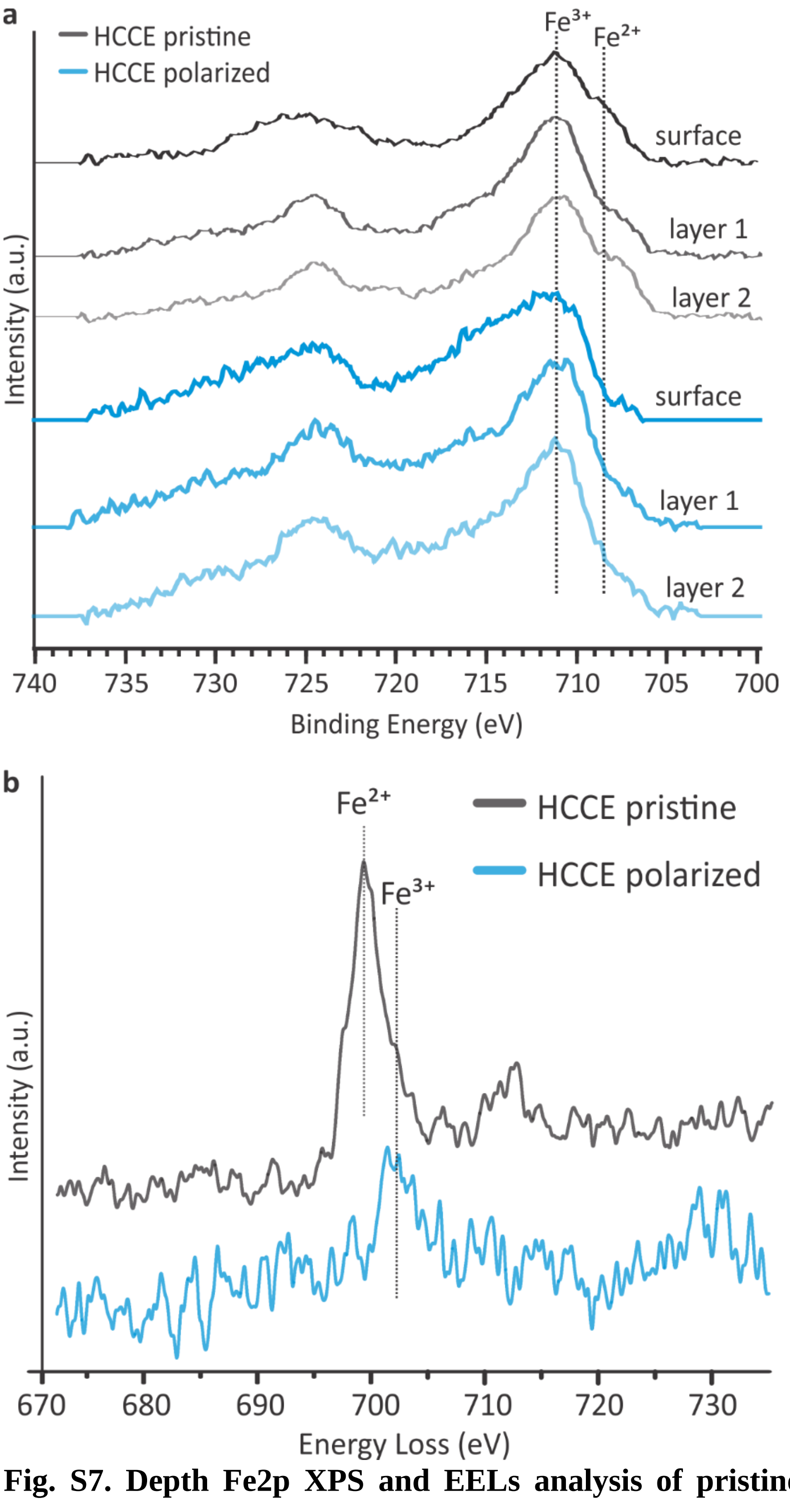

Fig. S7. Depth Fe2p XPS and EELs analysis of pristine and polarized HCCE. (**a**) High resolution Fe2p XPS depth profile spectra and **(b)** EELS spectra of pristine (black) and polarized (blue) HCCE electrodes

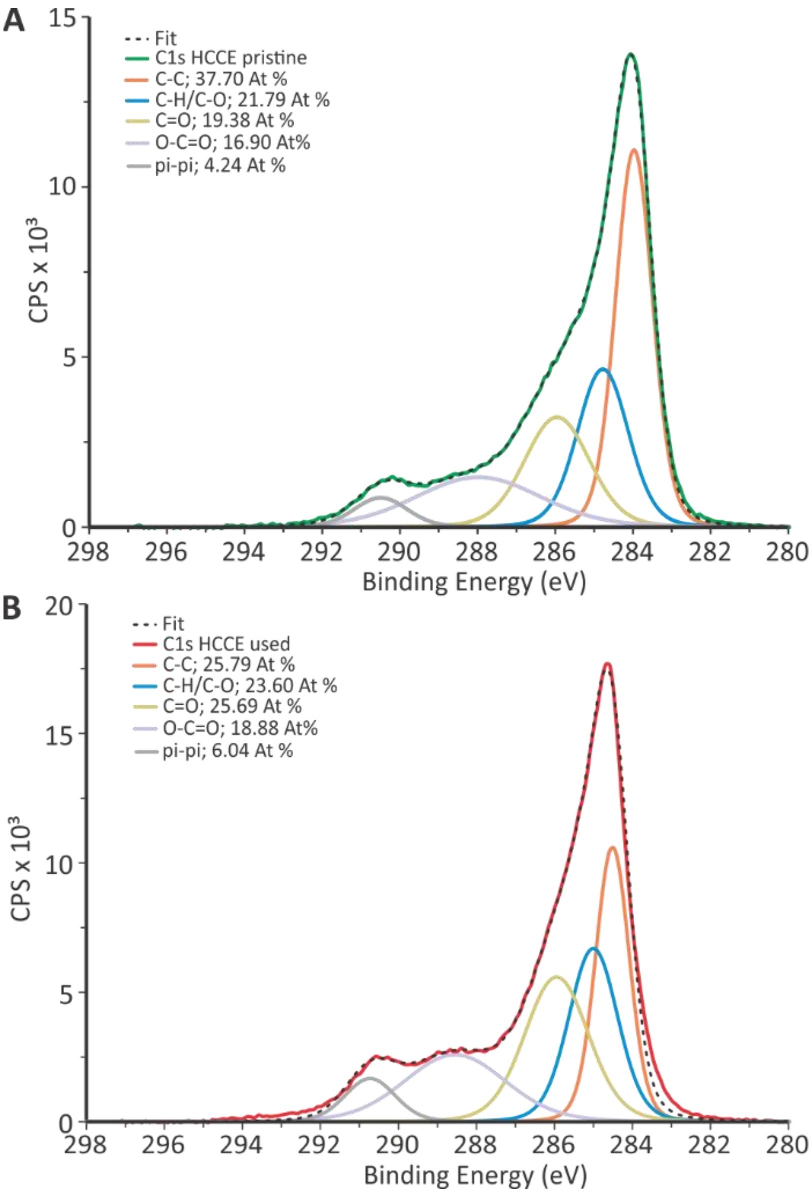

Fig. S8. Fitted high resolution C1s spectra.
(**a**) Fitted C1s high resolution spectra of pristine HCCE; fit (black dotted), C1s pristine HCCE (green), C-C (orange), C-H/C-O (blue), C=O (yellow), O-C=O (light purple), and pi-pi (gray). (**b**) Fitted C1s high resolution spectra of polarized HCCE; fit (black dotted), C1s polarized HCCE (red), C-C (orange), C-H/C-O (blue), C=O (yellow), O-C=O (light purple), and pi-pi (gray).

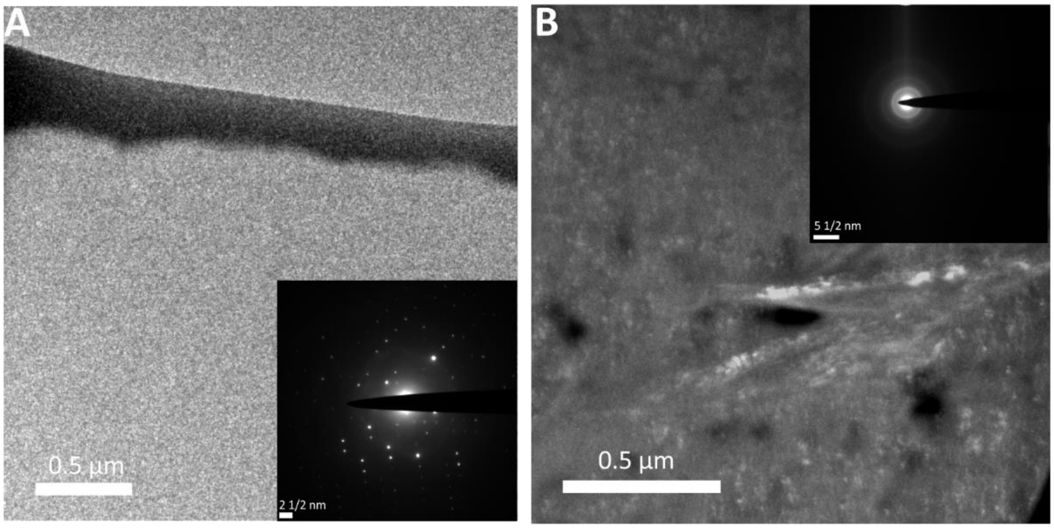

Fig S9. HR-TEM images of pristine (A) and polarized (B) HCCE electrodes.

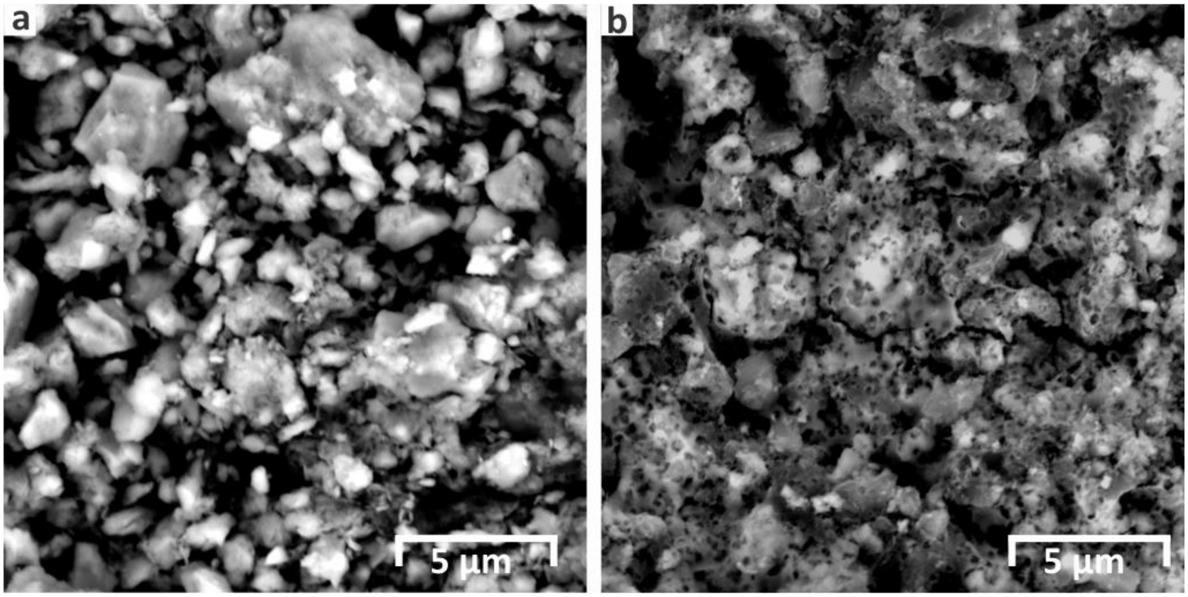

Fig S10. Pristine and polarized HCCE SEM images. SEM images showing (**a**) SEM image of pristine HCCE. (**b**) SEM image of HCCE after galvanostatic polarization highlighting the change in particle size and porosity of particles after anodic polarization.

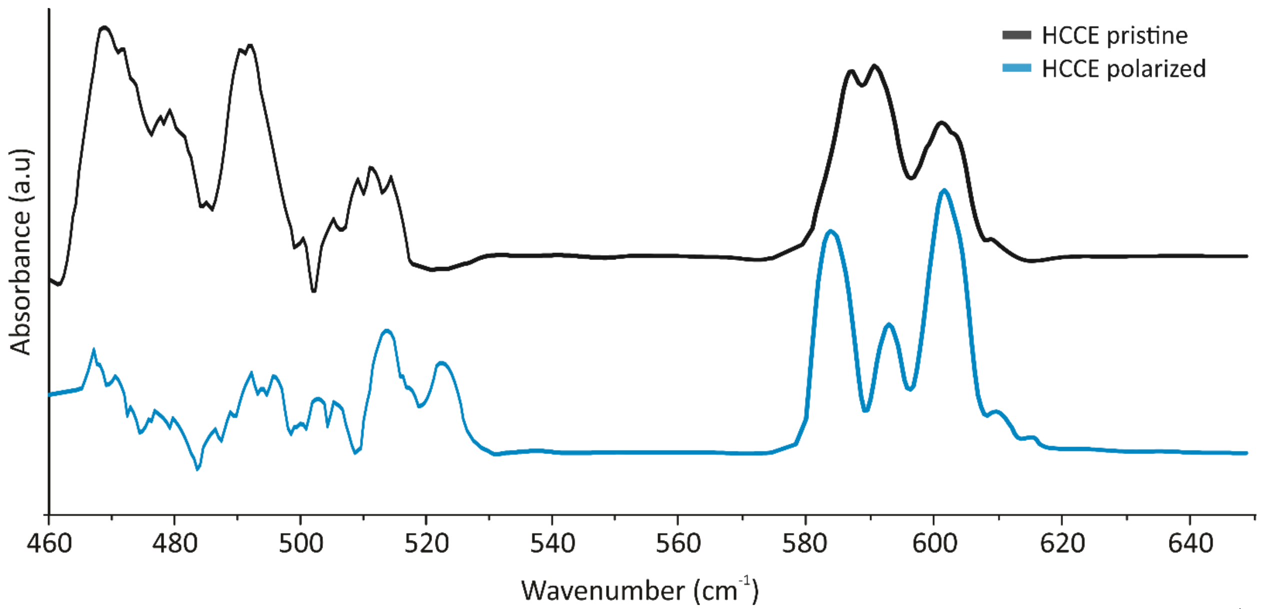

Fig. S11. FTIR spectra of polarized (blue) and pristine (black) HCCE between 460 and 640 cm^{-1}.

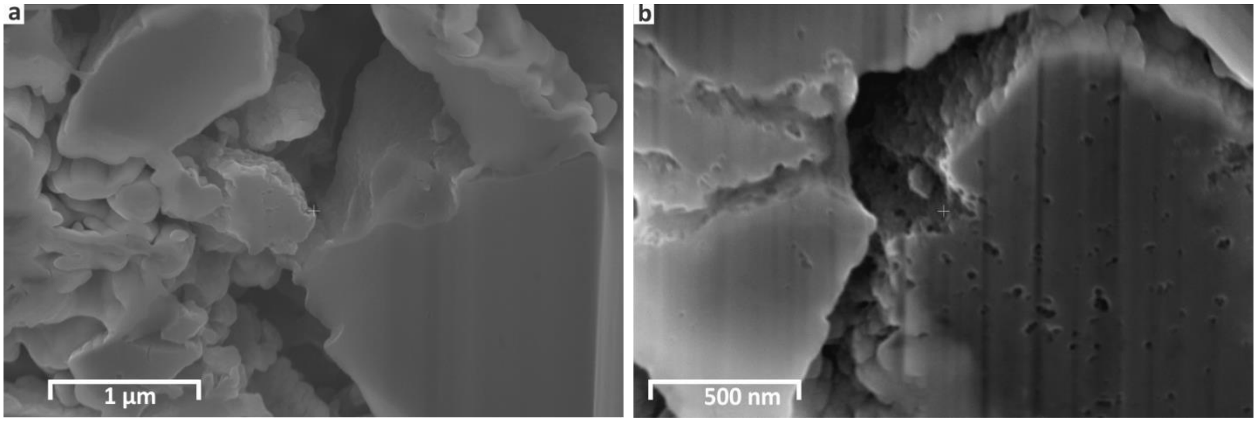

Fig S12. Pristine and polarized HCCE SEM-FIB images. Showing pristine HCCE (**a**) and polarized HCCE (**b**) with extensive nanopore formation.

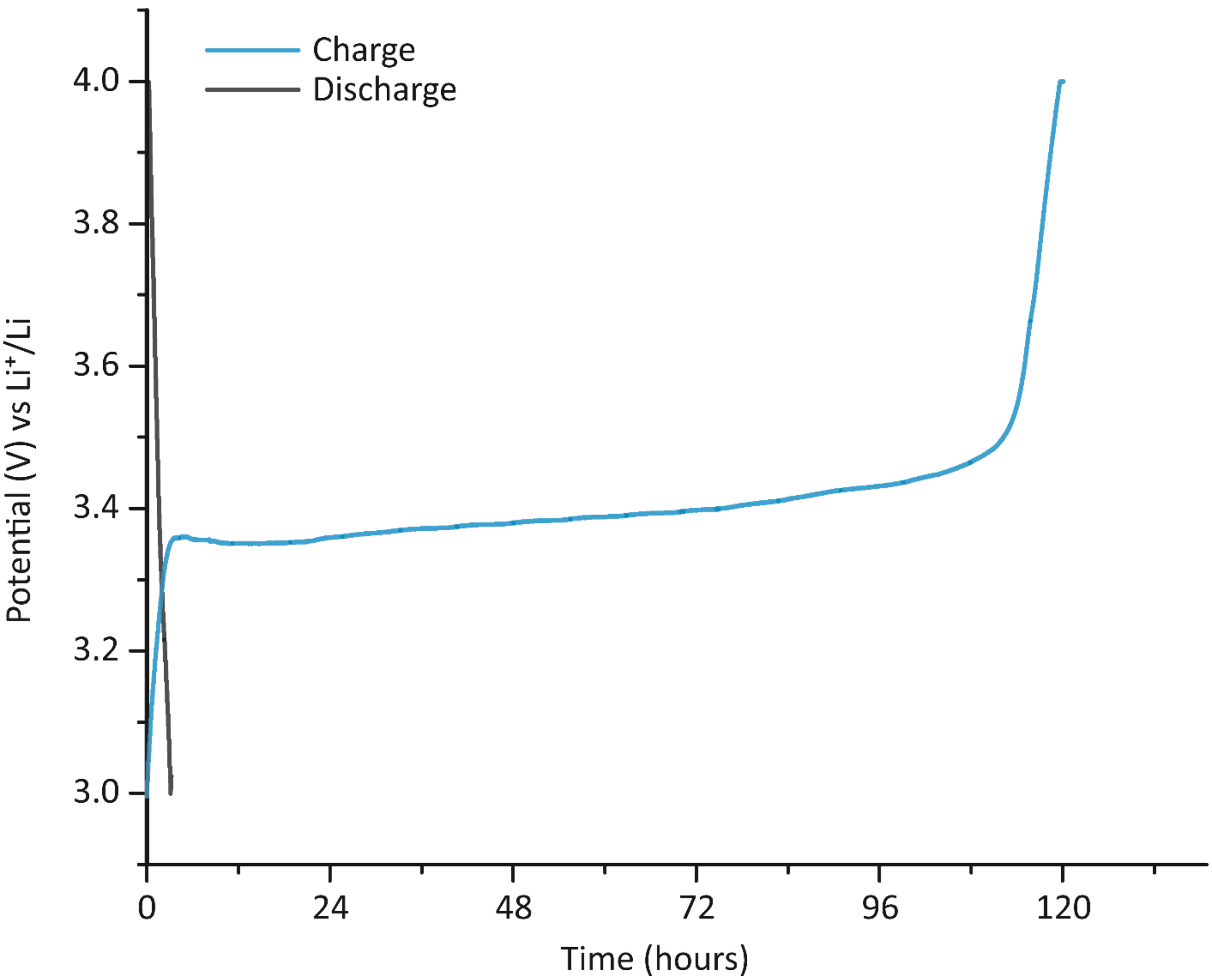

Fig S13. HCCE GCPL with 4.0 V vs Li^+/Li potential limit.

Supplemental Note: To probe the indemnity of the deintercalated metal ion and to interpret whether structural degradation is due to ion deintercalation or reaction with oxidized electrolyte, we performed GCPL with a potential limit of 4.0 V to only include iron oxidation and avoid any structural reactions with oxidized electrolyte at higher voltages (e.g. 4.3 V). Upon discharging, we see the same rapid capacitive discharge behavior as originally reported in Fig 2C, suggesting that the charging process altered the structure and that deintercalation of lithium was not possible, thus we can confirm that lithium is deintercalated since the structure would be able to reversibly discharge if lithium had not been removed from the structure.

Table S1. Crystal structure parameters of hectorite

**Crystal structure and parameters**					
Crystal system	a_0 (Å)	b_o (Å)	c_0 (Å)	α (°)	β (°)
Monoclinic	5.2401	9.0942	10.7971	90	99.21

Hectorite's CIF file was downloaded from the American Minerologist Crystal Structure Database, database code: amcsd 0015819. Cesium interplanar ions were replaced with sodium ions. Iron was added as an octahedral ion and occupancies of Mg, Fe, and Li were then adjusted based off our ICP-MS analysis elemental ratios.

Table S2. ICP-MS elemental composition of Hectorite gangue

**Metal Oxide**	**Metal Oxide %**
CaO	3.20
Fe_2O_3	3.53
Li_2O	0.50
MgO	5.64
Na_2O	4.73
K_2O	1.40
Al_2O_3	4.65
B_2O_3	12.8
Other minority metal oxides (cumulative)	0.68
Indigestible mass (quartz)	62.87

Table S3. ICP-MS quantification of pristine and polarized HCCEs

**Element**	**Pristine HCCE**	**Polarized HCCE**	**Amount removed**
	**moles ($x10^{-3}$)**	**moles ($x10^{-3}$)**	**(%)**
Ca	57.1	11.6 ± 2.5	79.6 ± 4.5
Na	152.6	40.26 ± 4.1	73.6 ± 2.6
Mg	139.8	40.7 ± 3.5	70.9 ± 2.5
Li	30.3	14.93 ± 1.3	50.7 ± 4.4
Fe	44.2	42.3 ± 2.7	4.4 ± 6.1

Standard deviations are given as 3σ, or 99.97 % certainty.

Table S4. Energy and CO_2 intensity of incumbent LGR process.

LGR Extraction plant overview			
**Chemical**	**t used / t LCE [a]**	**t CO_2 / t of chemical [b]**	**t CO_2 / t LCE**
H_2SO_4	1.67	0.037	0.062
$CaCO_3$	6.15	0.009	0.055
$CaSO_4$	14.68	0.004	0.058
NaOH	0.46	1.9	0.874
$Al_2(SO_4)_3$	0.63	0.49	0.308
**Power consumption**	**MWh / t LCE [a]**	**t CO_2 / MWh [b]**	**t CO_2 / t LCE**
Extraction plant	12.1	0.453 (distributed Natural gas)	5.47 (distributed Natural gas)
		1.05 (distributed coal)	12.73 (distributed coal)
		0.252 (distributed CA mix grid)	3.04 (distributed CA mix grid)
**Total – distributed natural gas**			**6.83**
**Total – distributed coal**			**14.1**
**Total – distributed mixed CA grid**			**4.40**

[a]: Values obtained from reference 65 in main manuscript.

[b]: Values obtained from GREET version 2023[3]

Supplementary References

1 Zhang, H. *et al.* Direct extraction of lithium from ores by electrochemical leaching. *Nature Communications* 15, 5066, doi:10.1038/s41467-024-48867-0 (2024).

2 Marcandalli, G., Goyal, A. & Koper, M. T. M. Electrolyte Effects on the Faradaic Efficiency of CO2 Reduction to CO on a Gold Electrode. *ACS Catalysis* 11, 4936-4945, doi:10.1021/acscatal.1c00272 (2021).

3 Greenhouse gases, Regulated Emissions, and Energy use in Technologies Model ® (2023 Excel) (United States, 2023).
