# Supplementary material for: Electrochemical lithium extraction from hectorite ore
Source: Commun Chem. 2024 Dec 3;7:285. doi: 10.1038/s42004-024-01378-x (PMC11614861; doi:10.1038/s42004-024-01378-x)
Supplement: Supplementary file 2 — Description of Additional Supplementary Files [file 42004_2024_1378_MOESM2_ESM.pdf]

**Description of Additional Supplementary Files**

File name- Supplementary Data 1

File description- Supplementary Data for Figures 2A-2D and Figures 3A-3D.
